# Influence of gestational history on neural tube defects and Eisenmenger syndrome: associations with intellectual disability and visual impairment in children and adults – a systematic review

**DOI:** 10.3389/fped.2025.1522839

**Published:** 2025-11-26

**Authors:** Dianasari, Chamelia Rohadatul ‘Aissy, R. Mohamad Javier, Gabriela Nativity, Syarif Syamsi Ahyandi, Siti Hani Amiralevi, Faisal Gani Putra Arlond, Khomariyana Purnama Sari, Mohammad Reza Riandata, Crysciando Jefryco Putra, Christopher Bryant Prathama, Fellicia Raphaela Thiono, Januar Ishak Hutasoit, Didik Setiyadi, Aflah Al Faiyq, Andika Prasetyo Arifin, Bernadetha Kusuma Kris Firmantya Tei Seran, David Panahatan, Muhammad Abni Setiawan, Fairuz Rifani, Afif Ferdian, Kenty Regina, Dania, Fatih Farabi, Georaldhy Yussufy Caecarma, Jonathan Alvin Wiryaputra, Auliya Yudia Yasyfin, M. Izdad Irfani Fanada, Laksmitha Saktiono Safitri, Chabib Fachry Albab, Basyar Adnani, Muhammad Rizky Hidayat, Maghfira Dwivani Rahmaputri, Trivena Sutarsa, Leony Octavia, Meliani Fransiska Andita, Harsya Parma Phastika, Johannes Tanaka, Dewi Sekarsari, Dita Ayu Dewi Laras Sati, Musthofa Chandra Ramabuana, Rilianda Simbolon, M. Rizki Fazrian Danu, Aulia Rachman, Fachira Rachel Agfata, Stefany Palyama, Andra Purwanto Yogatama Putra, Abraham Emzura Mamanta Sitepu, Restiko Maleo Fibullah, Adistia Maulidiah, Aulia Syifa, Subandono Bambang Indrasto, Abdul Alim, Renan Sukmawan, Rachmanto, Anastasia Asylia, Hayatun Nufus, Pertiwi Febriana Chandrawati, Aan Dwi Prasetyo, Lucky Sutanto, Moch. Aleq Sander

**Affiliations:** 1 RSUD Dr. Soetomo Surabaya, Surabaya, Indonesia; 2 RSAU dr. Moenir, Malang, Indonesia; 3 RS Dr. Cipto Mangunkusumo, Central Jakarta, Indonesia; 4 RSUD dr. Doris Sylvanus Kalimantan Tengah, Palangka Raya, Indonesia; 5 RSIA Soerya, Sidoarjo, Indonesia; 6 RSAU Dr. M. Sutomo Kubu Raya, Kubu Raya, Indonesia; 7 Bali International Hospital, Denpasar, Indonesia; 8 RSM Siti Khodijah Gurah, Kediri, Indonesia; 9 RSI Sakinah Mojokerto, Mojokerto, Indonesia; 10 RSUD Ploso, Jombang, Indonesia; 11 RSUD Ciamis, Ciamis, Indonesia; 12 RSUS Siloam Karawaci, Tangerang, Indonesia; 13 Pusat Jantung Nasional Harapan Kita Jakarta, West Jakarta, Indonesia; 14 Arsadia Medika Clinic, Bogor, Indonesia; 15 RSUD Jombang, Jombang, Indonesia; 16 RS QADR Kabupaten Tangerang, Tangerang, Indonesia; 17 RS Fastabiq Sehat PKU Muhammadiyah Pati, Pati, Indonesia; 18 RS UKI Jakarta, East Jakarta, Indonesia; 19 RSU Haji Surabaya, Surabaya, Indonesia; 20 RS Tk II Dustira, Cimahi, Indonesia; 21 RSU Permata Bunda Malang, Malang, Indonesia; 22 RS Sentra Medika Cibinong, Cibinong, Indonesia; 23 Fakultas Kedokteran Universitas Sebelas Maret, Surakarta, Indonesia; 24 Heartology Cardiovascular Hospital, South Jakarta, Indonesia; 25 RS Universitas Airlangga Surabaya, Surabaya, Indonesia; 26 RSU PKU Muhammadiyah Delanggu, Klaten, Indonesia; 27 RS PKU Muhammadiyah Gubug, Grobogan, Indonesia; 28 RS Dr Tadjuddin Chalid, Makassar, Indonesia; 29 Fakultas Kedokteran Universitas Padjajaran, Sumedang, Indonesia; 30 Fakultas Kedokteran Universitas Tarumanagara, West Jakarta, Indonesia; 31 RSUD Kalisat, Jember, Indonesia; 32 RS Mitra Medika Pontianak, Pontianak, Indonesia; 33 RS Pertamina Balikpapan, Balikpapan, Indonesia; 34 RSU Regina Maris, Medan, Indonesia; 35 RSUD Margono Soekarjo, Purwokerto, Indonesia; 36 RS Sariasih Sangiang, Tangerang, Indonesia; 37 RSU Cut Meutia Aceh Utara, Lhokseumawe, Indonesia; 38 RSUD Dr. H Soewondo, Kendal, Indonesia; 39 RS Prasetya Husada, Malang, Indonesia; 40 RS Parindu, Sanggau, Indonesia; 41 RSUD Kayuagung, Kayuagung, Indonesia; 42 RSUD Demang Sepulau Raya, Lampung, Indonesia; 43 RSPAD dr. Gatot Soebroto, Central Jakarta, Indonesia; 44 RS Persahabatan, East Jakarta, Indonesia; 45 RS Siloam Agora, Central Jakarta, Indonesia; 46 RSUP Dr. Sardjito Yogyakarta, Sleman, Indonesia; 47 RS Universitas Muhammadiyah Malang, Malang, Indonesia; 48 RS Kartika Husada Tanjungpura, Pontianak, Indonesia

**Keywords:** gestation, neural tube defect, Eisenmenger syndrome, intellectual disability, visual eye disease, systematic review

## Abstract

The period between conception and childbirth is known as gestational time. The condition of the baby can be affected by processes involved in pregnancy and delivery. History of diabetes and obesity during pregnancy has been proven to increase the risk of Eisenmenger Syndrome and Neural Tube Defect. Eisenmenger Syndrome is a congenital heart anomaly. Congenital heart disease, leading to circulation problems, includes issues with the iris stromal blood vessels, a characteristic feature of Eisenmenger syndrome. The dilation of iris stromal blood vessels due to this issue may lead to visual impairment/disorders. Additionally, individuals with Down syndrome and other forms of mental disorders suffer from congenital heart diseases. To analyze the influence of gestational history on the occurrence of neural tube defects and Eisenmenger syndrome, accompanied by intellectual disability and visual impairment in children and adults. A systematic review in this study was constructed using the Preferred Reporting Items for Systematic Reviews and Meta-Analyses (PRISMA) technique. This approach ensures that all stages and research procedures are systematically followed. A total of 2,327 results were obtained after collecting sources from Google Scholar and articles published between 2018 and 2023 were filtered with defined inclusion and exclusion criteria to ensure appropriate study selection. The classification of the number of Scopus-indexed journals is as follows: the number of Q2 Scopus-indexed journals is 5, with a total of five journals taken and used as references for systematic observational research. Gestational history significantly influences the occurrence of neural tube defects and Eisenmenger syndrome, accompanied by intellectual disability and visual impairment in children and adults.

## Introduction

Pregnancy represents a complex physiological state in which fetal development is exquisitely sensitive to maternal metabolic and environmental conditions. The gestational period—from conception to birth—is a critical window for organogenesis, including neural tube closure and cardiac morphogenesis. Deviations in maternal homeostasis, such as hyperglycemia, obesity, and nutritional imbalances, have been consistently implicated in the disruption of embryonic signaling pathways, thereby increasing the risk of congenital anomalies such as neural tube defects (NTDs) and Eisenmenger syndrome (ES) (1, 2).

NTDs, including spina bifida and anencephaly, result from the failure of the neural tube to close around the fourth week of gestation. Their molecular etiology involves dysregulation in folate-dependent one-carbon metabolism, oxidative stress, impaired DNA methylation, and abnormal Wnt/Notch signaling—all of which are essential to early neurodevelopment (3, 4). Maternal obesity and pregestational diabetes further elevate NTD risk, potentially through hyperglycemia-induced embryopathy and lipid signaling dysregulation via mTOR and SREBP pathways (1).

Eisenmenger syndrome, in contrast, emerges from uncorrected congenital heart defects such as ventricular septal or atrioventricular septal defects, which ultimately lead to pulmonary arterial hypertension and right-to-left shunting (5). The underlying pathophysiology is marked by progressive pulmonary vascular remodeling, endothelial dysfunction, and chronic hypoxemia, with molecular contributions from disrupted TGF- β/BMP signaling and oxidative stress pathways (6). Maternal hyperglycemia and metabolic syndromes during pregnancy may influence the expression of cardiogenic transcription factors (e.g., GATA4, NKX2-5), thereby predisposing the fetus to structural cardiac anomalies (7). These anomalies often coexist with neurodevelopmental and sensory deficits. Intellectual disability (ID) is commonly observed in individuals with congenital heart disease, particularly in syndromic conditions like Down syndrome, where trisomy 21 disrupts both neurogenesis and cardiogenesis (8). Furthermore, ES-related hypoxemia is associated with cerebral microvascular changes that may impair cognition.

Visual dysfunction in ES is another significant concern. Chronic hypoxemia and erythrocytosis can lead to retinal and episcleral vessel dilation, and iris microhemangiomas, resulting in non-traumatic hyphema and vision impairment (9). These ocular pathologies have been reported in both pediatric and adult populations affected by ES.

Despite emerging insights, there remains a scarcity of integrative reviews that address the molecular, clinical, and psychosocial dimensions linking gestational risk factors to these congenital conditions. This systematic review aims to address this gap by synthesizing current evidence on the influence of gestational history on NTD and ES, and their associations with intellectual disability and visual impairment, with implications for prevention, management, and future therapies.

## Materials and methods

**Figure 1:**
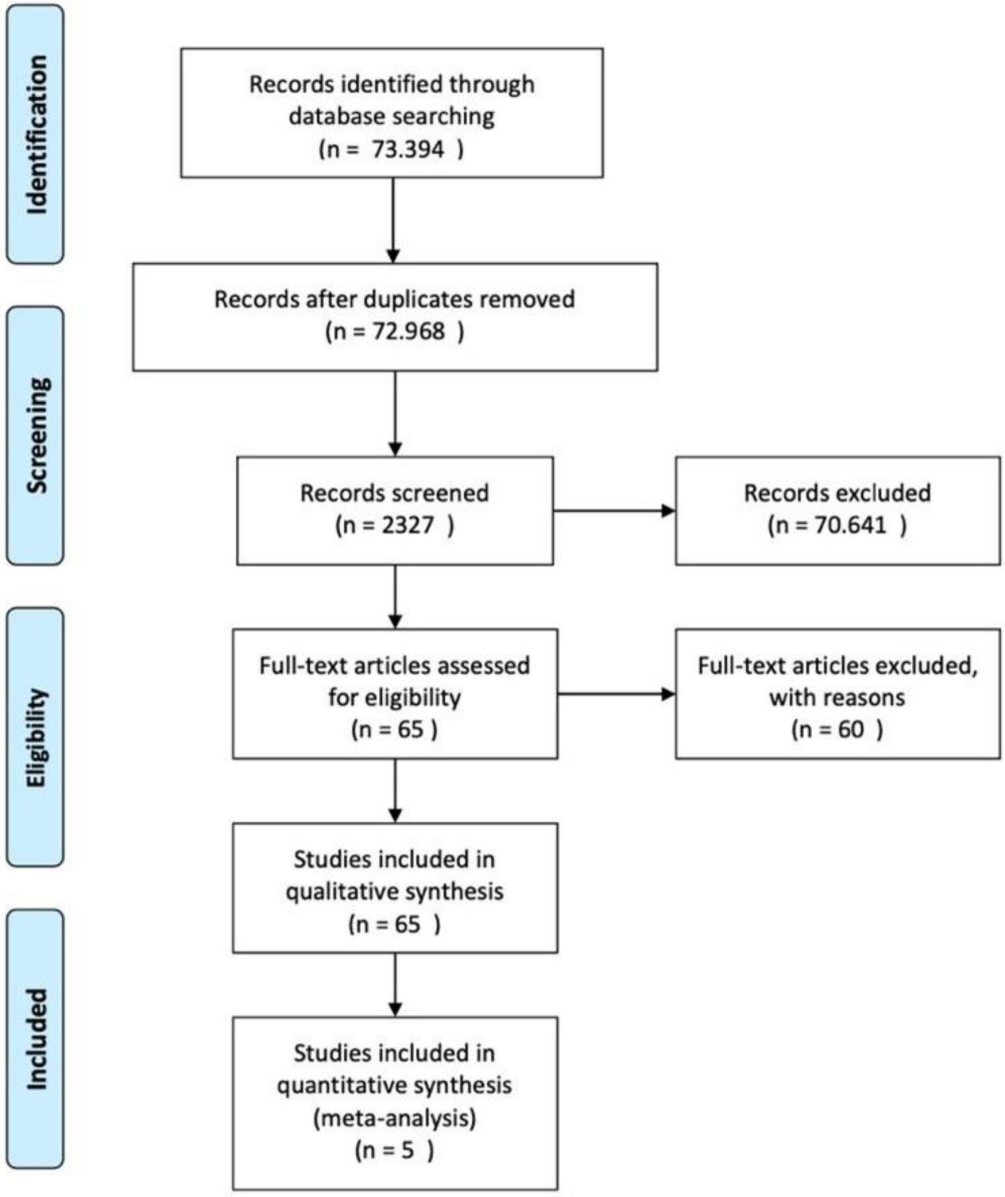
Preferred reporting items for systematic reviews and meta-analyses (PRISMA) statement of the systematic review.

This research employs a systematic review approach following the Preferred Reporting Items for Systematic Reviews and Meta-analysis (PRISMA) guidelines for systematic reviews (Figure 1). This method is systematically executed, adhering to correct research steps and protocols. A systematic review is a method that classifies and categorizes previously generated evidence through a structured series of evaluation, review, and classification. The systematic review procedure involves thorough preparation and sequencing, distinguishing it significantly from methods that merely provide literature studies.

The methodological quality of each included study was appraised using the GRADE approach (10) complemented by the Oxford Centre for Evidence-Based Medicine Levels of Evidence (Oxford 2011). Two reviewers independently assessed risk of bias, indirectness, inconsistency, imprecision and publication bias. Discrepancies were resolved by consensus with a third reviewer. Studies were then classified as High, Moderate, Low, or Very Low confidence (GRADE) and assigned an Oxford level (1-5). The resulting grades are presented in Table 1 and were used to weight the synthesis of findings.

**Table 1:** Resulting GRADES of included study.

Study (author, year)	Design/population	GRADE confidence	Oxford level	Key findings (as in original)
Linnell et al. 2022 (3)	Human observational (prospective cohort)	Moderate (observational, limited confounding control)	2b (individual cohort)	Obese mothers → sub-optimal folate → higher NTD risk
Zhang et al. 2023 (1)	Animal experiment (FVB & NOD mice)	Very Low (pre-clinical, indirect to human outcome)	5 (mechanistic/*ex vivo*)	Lipid accumulation + high-fat diet → yolk-sac abnormalities
Mohamed et al. 2022 (6)	Human screening study (cross-sectional)	Low (screening accuracy, no longitudinal follow-up)	2c (outcome research)	Pulse oximetry efficiently detects CHD early
Santoro & Steffensen 2021 (8)	Review of CHD in Down syndrome (case-series synthesis)	Low (narrative review, potential selection bias)	4 (case-control/series)	CHD prevalence 0-58%; strong link with ID
Ison et al. 2022 (9)	Case report (single patient)	Very Low (single-case, no comparators)	5 (expert opinion)	Iris hemangioma & retinal dilation → vision loss in ES

The systematic review comprises the following stages: 1) Developing the Background and Objectives of the Study; 2) Identification of problems; 3) Literature search; 4) Study selection; and 5) Quality Assessment. 6) Data synthesis.

### Developing the background and objectives of the study

The initial step in the systematic review involves crafting the background and study objectives. In this context, the systematic review addresses the impact of gestational history on the occurrence of neural tube defects and Eisenmenger syndrome, along with intellectual disability and visual impairment in both children and adults.

### Identification of problems

The research delves into existing research journals, identifying issues related to the impact of gestational history on the occurrence of neural tube defects and Eisenmenger syndrome, accompanied by intellectual disability and visual impairment in children and adults.

### Literature search

Utilizing easily accessible journal portals like MEDLINE and Google Scholar, the researcher conducted a targeted search using following search phrase in combination: Gestational history OR pregnancy history; neural tube defect OR neural tube malformation; eisenmenger syndrome OR eisenmenger complex OR eisenmenger disease; intellectual disability OR cognitive impairment; visual eye disease OR visual impairment. The filters English language, year = “2018–2023”, and journal articles or observational studies or randomised controlled trial (RCTs) were applied. The journal search was conducted in January 2024. It yielded 73,394 journal articles in total.

### Study selection

Before abstracts were screened, duplicates were removed. Five independent reviewers screened the titles, abstracts and full texts, differences were resolved by consensus. Studies were included according to the predetermined inclusion/exclusion criteria. For inclusion criteria, we considered RCTs, observational, case report and cohort studies published in peer-reviewed journals, journals published between 2018 and 2023, with research articles focusing on the occurrence of neural tube defects and Eisenmenger syndrome, accompanied by intellectual disability and visual impairment in both children and adults, the independent variable studied in these articles is gestational history, and only articles indexed by Scopus were included. Conversely, exclusion criteria involved articles with incomplete texts, those not published in journals, or not addressing the specified dependent variables. Additionally, articles that couldn't be accessed in their entirety were excluded from consideration. Additionally, only journals indexed by Scopus within the Q1-Q4 quartiles were included, with a focus on content directly relevant to investigating the impact of gestational history on the specified conditions. This meticulous selection process aimed to enhance the quality and relevance of the studies incorporated into the systematic review. Following the screening process, relevant data was extracted from the selected articles. The data extraction stage involved evaluating the five chosen articles, noting their main conclusions for subsequent data synthesis.

### Data synthesis

Given the heterogeneity in study designs, populations, and outcome measures, heterogeneity was assessed qualitatively by evaluating differences in study characteristics such as study design, sample size, outcome definitions, and measurement methods. Due to this heterogeneity, a formal quantitative meta-analysis was not feasible. Consequently, a narrative synthesis approach was employed to summarize and interpret the findings across studies. Structured tabulation of key study characteristics and outcomes was performed to aid synthesis. No subgroup analyses were conducted, as heterogeneity and data limitations precluded meaningful stratification. The methodological limitations and heterogeneity are discussed as potential sources of bias and constraints on evidence synthesis. The exclusion of inaccessible full texts and non-English studies was acknowledged as potential limitations that could introduce publication bias.

## Results

A total of 73,394 articles were identified through initial database searches. After removal of duplicates and eligibility screening, 5 high-quality studies indexed in Scopus Q2 journals met all inclusion criteria and were included in the final synthesis. The study selection process is summarized in Figure 1 (PRISMA flow diagram).

### Characteristics of included studies

**Figure 2:**
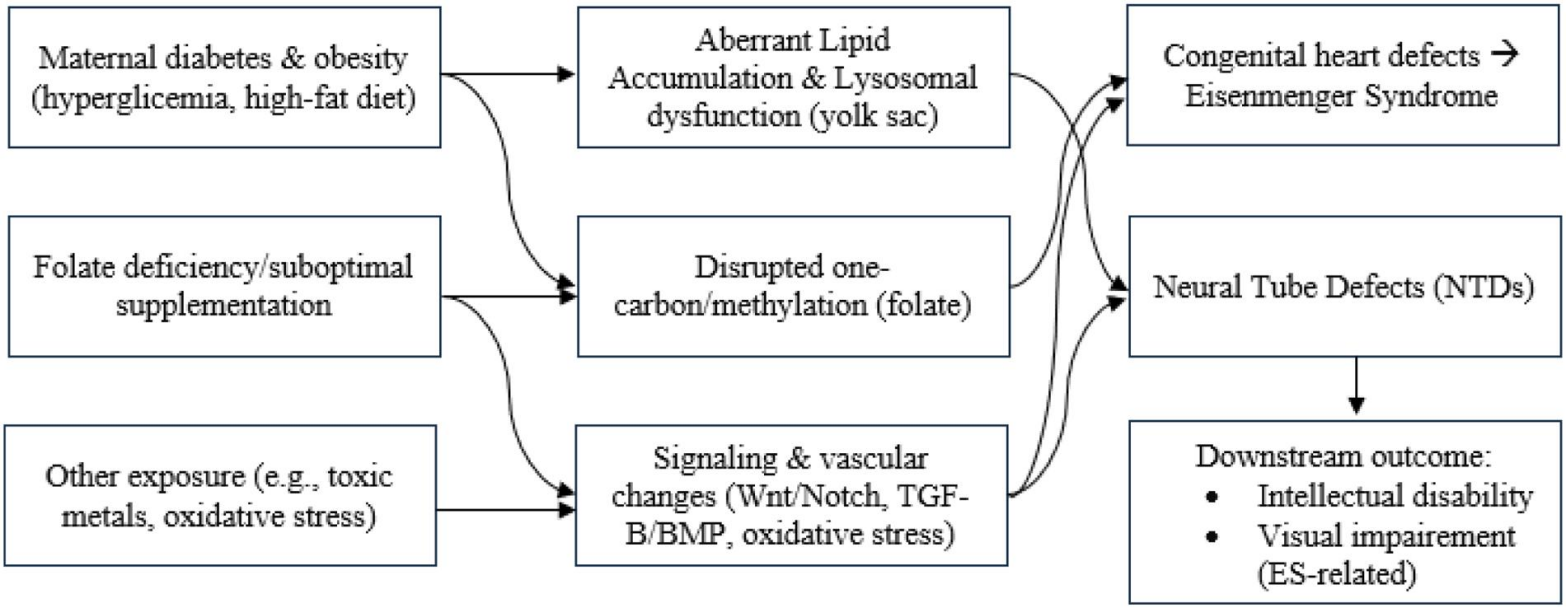
Conceptual map.

The five studies represented various methodological designs including prospective observational studies, a case report, a literature review, and animal-based mechanistic research. These studies focused on gestational risk factors, neural and cardiac developmental anomalies, and associated neuro-ocular outcomes (Table 2). Figure 2 showing conceptual map how gestational factors relate to each condition.

**Table 2:** Study included in the systematic review.

Study (author, year)	Design	Sample size/population	Setting	Duration	Key findings/outcomes
Linnell et al. 2022 (3)	Prospective observational study	328 pregnant women ≤18 weeks gestation	Antenatal clinics, UK	Not specified (data during early gestation)	Folic acid intake adequate in non-obese women; suboptimal in obese women → ↑ NTD risk
Zhang et al. 2023 (1)	Laboratory mechanistic study (animal model)	Inbred FVB and NOD mice (5–8 weeks old)	Laboratory, USA	Not specified	Maternal diabetes and obesity → abnormal lipid accumulation → NTD and CHD risk
Mohamed et al. 2022 (6)	Prospective screening study	460 newborns (Benha Univ. Hospital)	Egypt	July–Dec 2021	Pulse oximetry effective for early CHD detection
Santoro & Steffensen 2021 (8)	Systematic literature review	56 articles (20%–58% CHD in Down Syndrome)	Multicenter (global review)	Multi-year review	CHD highly prevalent in Down Syndrome; strong ID association
Ison et al. 2022 (9)	Case report	1 female, 56 years old	UK hospital	Not applicable	Eisenmenger syndrome caused chronic hypoxemia → iris microhemangiomas → vision loss

### Human observational evidence

Linnell et al. (3) reported that obese mothers exhibited suboptimal folate supplementation, correlating with an increased risk of NTDs (GRADE = Moderate). Mohamed et al. (6) demonstrated that pulse oximetry reliably identified congenital heart defects in asymptomatic newborns (GRADE = Low). Santoro & Steffensen (8) reviewed CHD prevalence in Down-syndrome cohorts, noting a 0%–58% range and a strong association with intellectual disability (GRADE = Low).

### Neural tube defects and maternal conditions

Two studies (1, 3) emphasized that maternal obesity and diabetes significantly compromise neural tube closure via metabolic and inflammatory pathways. Abnormal lipid accumulation in the visceral yolk sac, coupled with folate insufficiency, was linked to impaired lysosomal activity and increased NTD risk in murine models.

### Eisenmenger syndrome and fetal cardiac development

Findings from Mohamed et al. (6) and Santoro and Steffensen (8) reinforced that congenital heart defects are prevalent in infants born to mothers with poor metabolic regulation or syndromic genetic backgrounds. Prenatal conditions like hyperglycemia were shown to influence early cardiac septation, heightening the risk of postnatal Eisenmenger progression.

### Intellectual disability in congenital disorders

Santoro and Steffensen (8) reviewed literature showing that intellectual disability is highly prevalent in children with congenital heart disease, especially in Down syndrome, where altered neurodevelopmental signaling coexists with high CHD prevalence (20%–58%).

### Visual eye disease in Eisenmenger syndrome

The case study by Ison, Dorman and Imrie (9) provided clinical evidence linking retinal vascular changes and iris microhemangiomas to ES-related chronic hypoxemia. These vascular anomalies resulted in non-traumatic hyphema and visual impairment in the adult patient.

## Discussion

### Neural tube defects and gestational history: molecular insights

Neural tube defects (NTDs) arise from failure in neural tube closure during early embryogenesis, typically within the first 28 days post-conception. This process depends critically on folate-mediated one-carbon metabolism, which drives nucleotide biosynthesis, methylation reactions, and cellular proliferation (3). Maternal folate deficiency, hyperglycemia, and obesity disrupt this pathway, contributing to defective neural fold fusion and apoptosis regulation. Additionally, signaling pathways such as Wnt/β-catenin, Notch, and Sonic Hedgehog (SHH) are essential in neural tube patterning and are modulated by metabolic cues. In maternal diabetes and obesity, lipotoxicity and oxidative stress impair Wnt signaling and disrupt chromatin remodeling, increasing the risk for NTDs (1, 2).

### Eisenmenger syndrome: cardiac and vascular pathogenesis

Eisenmenger syndrome (ES) evolves from large, uncorrected congenital heart defects—most often ventricular septal defects (VSDs)—that lead to prolonged left-to-right shunting, followed by pulmonary hypertension and eventual shunt reversal. In-utero exposure to maternal hyperglycemia or hypoxia can interfere with cardiac looping and septation via dysregulation of NKX2.5, GATA4, and TBX5 gene expression (7). These transcription factors are critical in early cardiogenesis. Moreover, the TGF-β/BMP signaling axis, involved in vascular remodeling, becomes aberrant in ES, contributing to pulmonary arterial hypertension. Endothelial nitric oxide synthase (eNOS) dysfunction, mitochondrial ROS accumulation, and smooth muscle proliferation underlie the progressive vascular remodeling seen in ES patients (5).

### Intellectual disability and syndromic congenital heart disease

Intellectual disability (ID) is a frequent comorbidity in individuals with congenital heart defects, especially in syndromic contexts such as Down syndrome. Trisomy 21 results in overexpression of key genes (e.g., DYRK1A, RCAN1) involved in neural plasticity and oxidative stress regulation, compounding neurodevelopmental delays (8). Furthermore, in patients with Eisenmenger syndrome, chronic cerebral hypoxemia may impair neurogenesis, myelination, and synaptic development—exacerbating cognitive deficits across the lifespan.

### Eisenmenger syndrome and ocular sequelae

ES also affects the visual system due to sustained erythrocytosis and chronic hypoxemia, which drive dilation of the retinal and episcleral vasculature. This contributes to the formation of iris microhemangiomas, leading to complications like non-traumatic hyphema and gradual vision loss (9). These microvascular changes are thought to result from hypoxia-induced VEGF upregulation and impaired vascular autoregulation.

### Influence of gestational history on neural tube defect and Eisenmenger syndrome accompanied by intellectual disability and visual impairment in children and adults

The period from fertilization to childbirth, known as gestation, plays a pivotal role in fetal development. Gestational history, combined with other external variables, can lead to disturbances in fetal development, resulting in congenital anomalies. Neural Tube Defects (NTDs) and congenital heart defects are among the common anomalies. NTDs occur when the neural tube fails to close during embryonic development, and they are influenced by genetic and environmental factors. Eisenmenger Syndrome, characterized by congenital heart defects, negatively impacts circulatory functions, including issues with iris stromal blood vessels. These anomalies affect vision and can lead to vision loss. Cardiovascular diseases, especially congenital heart defects, are prevalent in individuals with Down Syndrome, contributing to adverse outcomes. Therefore, this study concludes that gestational history significantly influences the occurrence of neural tube defects and Eisenmenger Syndrome, accompanied by intellectual disability and visual impairment in both children and adults.

### Limitations of the study & medical implications

This review offers meaningful insights into the relationship between gestational history and the development of neural tube defects (NTDs) and Eisenmenger syndrome (ES), but several limitations must be acknowledged. Overall, the certainty of evidence linking adverse gestational exposures to NTDs and ES is limited (low to moderate). While observational studies provide consistent associations, the lack of high-certainty data (e.g., randomized controlled trials) restricts causal inference. Animal experiments generate plausible mechanistic pathways but, given their very-low confidence rating, should be interpreted as hypothesis-generating. The inclusion of only five eligible studies—spanning diverse designs such as observational research, case reports, and animal studies—limits generalizability and precludes meta-analysis due to methodological heterogeneity. Restricting the search to English-language, Scopus-indexed publications may introduce language and selection bias, while the absence of longitudinal or interventional data restricts causal interpretation and evidence on preventative strategies. Nonetheless, the findings underscore important clinical implications: optimizing maternal health through glycemic control, nutritional support (particularly folate), and weight management during pregnancy could significantly reduce the risk of NTDs and congenital heart defects. Furthermore, infants born with these conditions require early, multidisciplinary follow-up that includes neurodevelopmental and ophthalmologic surveillance to detect and manage intellectual disability and vision disorders. Future translational research should explore the modulation of shared molecular pathways—such as Wnt, TGF-β, oxidative stress, and folate metabolism—for novel therapeutic interventions during critical gestational periods.

## Conclusion

Gestational history exerts a profound influence on fetal development, with maternal metabolic disorders such as diabetes, obesity, and nutritional deficiencies increasing the risk for structural and functional anomalies. This review synthesizes evidence linking adverse gestational exposures to the onset of neural tube defects (NTDs) and Eisenmenger syndrome (ES)—conditions that significantly contribute to lifelong disability. The mechanisms underlying these associations involve disruption of folate-dependent metabolic pathways, oxidative stress, and altered Wnt, Notch, and TGF-β signaling, which impair neural tube closure and cardiac morphogenesis. In turn, these congenital anomalies are associated with downstream complications such as intellectual disability—notably in syndromic forms like Down syndrome—and visual impairments due to ES-related vascular changes. A proactive approach encompassing preconception care, maternal health optimization, early fetal anomaly detection, and postnatal neuro-ophthalmologic screening is vital. Future research should focus on molecular therapies targeting oxidative stress, epigenetic regulation, and vascular remodeling to mitigate the long-term burden of these developmental disorders.

## Data Availability

The raw data supporting the conclusions of this article will be made available by the authors, without undue reservation.
